# DUAL IMPACTS ON MEANING IN LIFE IN OLDER PERSONS VOLUNTEERING FOR ELDERLY MENTAL HEALTH PROGRAM

**DOI:** 10.1093/geroni/igz038.3549

**Published:** 2019-11-08

**Authors:** Dara K Y Leung, Joyce Sing, Lesley Sze, Jessica Tang, Shiyu Lu, Tianyin Liu, Gloria H Y Wong, Terry Lum

**Affiliations:** 1 The University of Hong Kong, Hong Kong, Hong Kong; 2 Department of Social Work and Social Administration, PokFuLam, Hong Kong

## Abstract

Volunteering provides sense of meaning in life. The impact of volunteering on different dimensions of meaning in life and the mechanisms explaining the effects have been rarely researched. This study examined the effects and the mechanism of a formal volunteering program for mental health in older persons, including training, service provision, and supervision, on two dimensions of meaning in life — presence of meaning and searching for meaning — among senior volunteers. A mixed method study was conducted. 103 volunteers (average age=63.3±6.6) completed assessments at three time points: before and after the training, and one-year after service provision. They self-assessed Meaning in Life Questionnaire (MLQ) and reported time use in different tasks. 26 of them participated in focus groups discussing their experience in the program. Volunteers’ search for meaning differed between time points (F(1.87,173.81)=3.20, p<.01) while presence of meaning persisted. Search for meaning reduced from before the training to after service provision (p<.05) as revealed by post-hoc tests. Proportion of home visit during service provision explained 2.7% of the variance of presence of meaning before and after service provision (R2=0.05, F(6,74)=1.376, p<.05). Findings from focus groups revealed that application of trained skills and building trusting relationship with their clients via home visits are sources of meaning. Formal volunteering may have dual impacts on meaning in life in older age: reducing search for meaning and maintaining presence of meaning. For senior volunteers, being able to apply what they learn and building social connects are the key factors for attaining meaning.

